# Supplementary material for: Review of Clinically Assessed Molecular Fluorophores for Intraoperative Image Guided Surgery
Source: Molecules. 2024 Dec 18;29(24):5964. doi: 10.3390/molecules29245964 (PMC11679787; doi:10.3390/molecules29245964)
Supplement: Supplementary file 1 [file molecules-29-05964-s001.zip › molecules-3341650-supplementary.pdf]

Supplementary Information

Table S1. Literature references of ICG used for sentinel lymph node (SLN) mapping

surgery type	References
breast cancer	Hojo, T.; Nagao, T.; Kikuyama, M.; Akashi, S.; Kinoshita, T. Evaluation of sentinel node biopsy by combined fluorescent and dye method and lymph flow for breast cancer. *Breast* **2010**, *19* (3), 210-213. DOI: 10.1016/j.breast.2010.01.014.<br>Kitai, T.; Kawashima, M. Transcutaneous detection and direct approach to the sentinel node using axillary compression technique in ICG fluorescence-navigated sentinel node biopsy for breast cancer. *Breast cancer* **2012**, *19* (4), 343-348. DOI: 10.1007/s12282-011-0286-1.<br>Sugie, T.; Sawada, T.; Tagaya, N.; Kinoshita, T.; Yamagami, K.; Suwa, H.; Ikeda, T.; Yoshimura, K.; Niimi, M.; Shimizu, A.; Toi, M. Comparison of the indocyanine green fluorescence and blue dye methods in detection of sentinel lymph nodes in early-stage breast cancer. *Annals of surgical oncology* **2013**, *20* (7), 2213-2218. DOI: 10.1245/s10434-013-2890-0.<br>Takeuchi, M.; Sugie, T.; Abdelazeem, K.; Kato, H.; Shinkura, N.; Takada, M.; Yamashiro, H.; Ueno, T.; Toi, M. Lymphatic mapping with fluorescence navigation using indocyanine green and axillary surgery in patients with primary breast cancer. *The breast journal* **2012**, *18* (6), 535-541. DOI: 10.1111/tbj.12004.<br>Hirche, C.; Murawa, D.; Mohr, Z.; Kneif, S.; Hunerbein, M. ICG fluorescence-guided sentinel node biopsy for axillary nodal staging in breast cancer. *Breast cancer research and treatment* **2010**, *121* (2), 373-378. DOI: 10.1007/s10549-010-0760-z.<br>Hutteman, M.; Mieog, J. S.; van der Vorst, J. R.; Liefers, G. J.; Putter, H.; Lowik, C. W.; Frangioni, J. V.; van de Velde, C. J.; Vahrmeijer, A. L. Randomized, double-blind comparison of indocyanine green with or without albumin premixing for near-infrared fluorescence imaging of sentinel lymph nodes in breast cancer patients. *Breast cancer research and treatment* **2011**, *127* (1), 163-170. DOI: 10.1007/s10549-011-1419-0.<br>van der Vorst, J. R.; Schaafsma, B. E.; Verbeek, F. P.; Hutteman, M.; Mieog, J. S.; Lowik, C. W.; Liefers, G. J.; Frangioni, J. V.; van de Velde, C. J.; Vahrmeijer, A. L. Randomized comparison of near-infrared fluorescence imaging using indocyanine green and 99(m) technetium with or without patent blue for the sentinel lymph node procedure in breast cancer patients. *Annals of surgical oncology* **2012**, *19* (13), 4104-4111. DOI: 10.1245/s10434-012-2466-4.<br>Mieog, J. S.; Troyan, S. L.; Hutteman, M.; Donohoe, K. J.; van der Vorst, J. R.; Stockdale, A.; Liefers, G. J.; Choi, H. S.; Gibbs-Strauss, S. L.; Putter, H.; et al. Toward optimization of imaging system and lymphatic tracer for near-infrared fluorescent sentinel lymph node mapping in breast cancer. *Annals of surgical oncology* **2011**, *18* (9), 2483-2491. DOI: 10.1245/s10434-011-1566-x.

	Wishart, G. C.; Loh, S. W.; Jones, L.; Benson, J. R. A feasibility study (ICG-10) of indocyanine green (ICG) fluorescence mapping for sentinel lymph node detection in early breast cancer. *European journal of surgical oncology : the journal of the European Society of Surgical Oncology and the British Association of Surgical Oncology* **2012**, *38* (8), 651-656. DOI: 10.1016/j.ejso.2012.05.007.<br>Guo, W.; Zhang, L.; Ji, J.; Gao, W.; Liu, J.; Tong, M. Breast cancer sentinel lymph node mapping using near-infrared guided indocyanine green in comparison with blue dye. *Tumour biology : the journal of the International Society for Oncodevelopmental Biology and Medicine* **2014**, *35* (4), 3073-3078. DOI: 10.1007/s13277-013-1399-2.<br>He, K.; Chi, C.; Kou, D.; Huang, W.; Wu, J.; Wang, Y.; He, L.; Ye, J.; Mao, Y.; Zhang, G. J.; et al. Comparison between the indocyanine green fluorescence and blue dye methods for sentinel lymph node biopsy using novel fluorescence image-guided resection equipment in different types of hospitals. *Translational research : the journal of laboratory and clinical medicine* **2016**, *178*, 74-80. DOI: 10.1016/j.trsl.2016.07.010.
gastric cancer	Tummers, Q. R.; Boogerd, L. S.; de Steur, W. O.; Verbeek, F. P.; Boonstra, M. C.; Handgraaf, H. J.; Frangioni, J. V.; van de Velde, C. J.; Hartgrink, H. H.; Vahrmeijer, A. L. Near-infrared fluorescence sentinel lymph node detection in gastric cancer: A pilot study. *World journal of gastroenterology* **2016**, *22* (13), 3644-3651. DOI: 10.3748/wjg.v22.i13.3644.<br>Chen, Q. Y.; Zhong, Q.; Liu, Z. Y.; Li, P.; Lin, G. T.; Zheng, Q. L.; Wang, J. B.; Lin, J. X.; Lu, J.; Cao, L. L.; et al. Indocyanine green fluorescence imaging-guided versus conventional laparoscopic lymphadenectomy for gastric cancer: long-term outcomes of a phase 3 randomised clinical trial. *Nature communications* **2023**, *14* (1), 7413. DOI: 10.1038/s41467-023-42712-6 From NLM Medline.
melanoma	Gilmore, D. M.; Khullar, O. V.; Gioux, S.; Stockdale, A.; Frangioni, J. V.; Colson, Y. L.; Russell, S. E. Effective low-dose escalation of indocyanine green for near-infrared fluorescent sentinel lymph node mapping in melanoma. *Annals of surgical oncology* **2013**, *20* (7), 2357-2363. DOI: 10.1245/s10434-013-2905-x.<br>van der Vorst, J. R.; Schaafsma, B. E.; Verbeek, F. P.; Swijnenburg, R. J.; Hutteman, M.; Liefers, G. J.; van de Velde, C. J.; Frangioni, J. V.; Vahrmeijer, A. L. Dose optimization for near-infrared fluorescence sentinel lymph node mapping in patients with melanoma. *The British journal of dermatology* **2013**, *168* (1), 93-98. DOI: 10.1111/bjd.12059.
vulvar cancer	Schaafsma, B. E.; Verbeek, F. P.; Peters, A. A.; van der Vorst, J. R.; de Kroon, C. D.; van Poelgeest, M. I.; Trimbos, J. B.; van de Velde, C. J.; Frangioni, J. V.; Vahrmeijer, A. L.; Gaarenstroom, K. N. Near-infrared fluorescence sentinel lymph node biopsy in vulvar cancer: a randomised comparison of lymphatic tracers. *BJOG : an international journal of obstetrics and gynaecology* **2013**, *120* (6), 758-764. DOI: 10.1111/1471-0528.12173.<br>Hutteman, M.; van der Vorst, J. R.; Gaarenstroom, K. N.; Peters, A. A.; Mieog, J. S.; Schaafsma, B. E.; Lowik, C. W.; Frangioni, J. V.; van de Velde, C. J.; Vahrmeijer, A. L. Optimization of near-infrared fluorescent sentinel lymph

	node mapping for vulvar cancer. *American journal of obstetrics and gynecology* **2012**, *206* (1), 89 e81-85. DOI: 10.1016/j.ajog.2011.07.039.<br>Crane, L. M.; Themelis, G.; Arts, H. J.; Buddingh, K. T.; Brouwers, A. H.; Ntziachristos, V.; van Dam, G. M.; van der Zee, A. G. Intraoperative near-infrared fluorescence imaging for sentinel lymph node detection in vulvar cancer: first clinical results. *Gynecologic oncology* **2011**, *120* (2), 291-295. DOI: 10.1016/j.ygyno.2010.10.009.
cervical cancer	Schaafsma, B. E.; van der Vorst, J. R.; Gaarenstroom, K. N.; Peters, A. A.; Verbeek, F. P.; de Kroon, C. D.; Trimbos, J. B.; van Poelgeest, M. I.; Frangioni, J. V.; van de Velde, C. J.; Vahrmeijer, A. L. Randomized comparison of near-infrared fluorescence lymphatic tracers for sentinel lymph node mapping of cervical cancer. *Gynecologic oncology* **2012**, *127* (1), 126-130. DOI: 10.1016/j.ygyno.2012.07.002.<br>Rossi, E. C.; Ivanova, A.; Boggess, J. F. Robotically assisted fluorescence-guided lymph node mapping with ICG for gynecologic malignancies: a feasibility study. *Gynecologic oncology* **2012**, *124* (1), 78-82. DOI: 10.1016/j.ygyno.2011.09.025.
endometrial cancer	Rossi, E. C.; Ivanova, A.; Boggess, J. F. Robotically assisted fluorescence-guided lymph node mapping with ICG for gynecologic malignancies: a feasibility study. *Gynecologic oncology* **2012**, *124* (1), 78-82. DOI: 10.1016/j.ygyno.2011.09.025.<br>Rossi, E. C.; Kowalski, L. D.; Scalici, J.; Cantrell, L.; Schuler, K.; Hanna, R. K.; Method, M.; Ade, M.; Ivanova, A.; Boggess, J. F. A comparison of sentinel lymph node biopsy to lymphadenectomy for endometrial cancer staging (FIRES trial): a multicentre, prospective, cohort study. *The Lancet. Oncology* **2017**, *18* (3), 384-392. DOI: 10.1016/s1470-2045(17)30068-2.
non-small cell lung cancer	Gilmore, D. M.; Khullar, O. V.; Jaklitsch, M. T.; Chirieac, L. R.; Frangioni, J. V.; Colson, Y. L. Identification of metastatic nodal disease in a phase 1 dose-escalation trial of intraoperative sentinel lymph node mapping in non-small cell lung cancer using near-infrared imaging. *The Journal of thoracic and cardiovascular surgery* **2013**, *146* (3), 562-570; discussion 569-570. DOI: 10.1016/j.jtcvs.2013.04.010.
oropharyngeal cancer	Bredell, M. G. Sentinel lymph node mapping by indocyanin green fluorescence imaging in oropharyngeal cancer - preliminary experience. *Head & neck oncology* **2010**, *2*, 31. DOI: 10.1186/1758-3284-2-31.
colon cancer	Hirche, C.; Mohr, Z.; Kneif, S.; Doniga, S.; Murawa, D.; Strik, M.; Hunerbein, M. Ultrastaging of colon cancer by sentinel node biopsy using fluorescence navigation with indocyanine green. *International journal of colorectal disease* **2012**, *27* (3), 319-324. DOI: 10.1007/s00384-011-1306-5.<br>Currie, A. C.; Brigic, A.; Thomas-Gibson, S.; Suzuki, N.; Moorghen, M.; Jenkins, J. T.; Faiz, O. D.; Kennedy, R. H. A pilot study to assess near infrared laparoscopy with indocyanine green (ICG) for intraoperative sentinel lymph node mapping in early colon cancer. *European journal of surgical oncology : the journal of the European Society of Surgical Oncology and the British Association of Surgical Oncology* **2017**, *43* (11), 2044-2051. DOI: 10.1016/j.ejso.2017.05.026.
colorectal cancer	Cahill, R. A.; Anderson, M.; Wang, L. M.; Lindsey, I.; Cunningham, C.; Mortensen, N. J. Near-infrared (NIR) laparoscopy for intraoperative

	lymphatic road-mapping and sentinel node identification during definitive surgical resection of early-stage colorectal neoplasia. *Surgical endoscopy* **2012**, *26* (1), 197-204. DOI: 10.1007/s00464-011-1854-3.
prostate cancer	Jeschke, S.; Lusuardi, L.; Myatt, A.; Hruby, S.; Pirich, C.; Janetschek, G. Visualisation of the lymph node pathway in real time by laparoscopic radioisotope- and fluorescence-guided sentinel lymph node dissection in prostate cancer staging. *Urology* **2012**, *80* (5), 1080-1086. DOI: 10.1016/j.urology.2012.05.050
lymph flow evaluation	Watanabe, J.; Ota, M.; Suwa, Y.; Ishibe, A.; Masui, H.; Nagahori, K. Evaluation of lymph flow patterns in splenic flexural colon cancers using laparoscopic real-time indocyanine green fluorescence imaging. *International journal of colorectal disease* **2017**, *32* (2), 201-207. DOI: 10.1007/s00384-016-2669-4
lymphedema management	Tan, I. C.; Maus, E. A.; Rasmussen, J. C.; Marshall, M. V.; Adams, K. E.; Fife, C. E.; Smith, L. A.; Chan, W.; Sevick-Muraca, E. M. Assessment of lymphatic contractile function after manual lymphatic drainage using near-infrared fluorescence imaging. *Archives of physical medicine and rehabilitation* **2011**, *92* (5), 756-764 e751. DOI: 10.1016/j.apmr.2010.12.027
lymphatic abnormalities	Burrows, P. E.; Gonzalez-Garay, M. L.; Rasmussen, J. C.; Aldrich, M. B.; Guilliod, R.; Maus, E. A.; Fife, C. E.; Kwon, S.; Lapinski, P. E.; King, P. D.; Sevick-Muraca, E. M. Lymphatic abnormalities are associated with RASA1 gene mutations in mouse and man. *Proceedings of the National Academy of Sciences of the United States of America* **2013**, *110* (21), 8621-8626. DOI: 10.1073/pnas.1222722110
squamous cell carcinoma	van den Berg, N. S.; Brouwer, O. R.; Klop, W. M.; Karakullukcu, B.; Zuur, C. L.; Tan, I. B.; Balm, A. J.; van den Brekel, M. W.; Valdes Olmos, R. A.; van Leeuwen, F. W. Concomitant radio- and fluorescence-guided sentinel lymph node biopsy in squamous cell carcinoma of the oral cavity using ICG-(99m)Tc-nanocolloid. *European journal of nuclear medicine and molecular imaging* **2012**, *39* (7), 1128-1136. DOI: 10.1007/s00259-012-2129-5

Table S2. Literature references of ICG for bile duct identification

surgery type	References
laparoscopic cholecystectomy	Schols, R. M.; Bouvy, N. D.; Masclee, A. A.; van Dam, R. M.; Dejong, C. H.; Stassen, L. P. Fluorescence cholangiography during laparoscopic cholecystectomy: a feasibility study on early biliary tract delineation. *Surgical endoscopy* **2013**, *27* (5), 1530-1536. DOI: 10.1007/s00464-012-2635-3. Schols, R. M.; Bouvy, N. D.; van Dam, R. M.; Masclee, A. A.; Dejong, C. H.; Stassen, L. P. Combined vascular and biliary fluorescence imaging in laparoscopic cholecystectomy. *Surgical endoscopy* **2013**, *27* (12), 4511-4517. DOI: 10.1007/s00464-013-3100-7. Jacqueline van den Bos; Rutger M Schols; Misha D Luyer; Ronald M van Dam; Alexander L Vahrmeijer; Wilhelmus J Meijerink; Paul D Gobardhan; Gooitzen M van Dam; Nicole D Bouvy; Stassen, L. P. S. Near-infrared fluorescence cholangiography assisted laparoscopic cholecystectomy versus

	conventional laparoscopic cholecystectomy (FALCON trial): study protocol for a multicentre randomised controlled trial *BMJ Open* **2016**, *6*, e011668. DOI: 10.1136/bmjopen-2016011668.<br>Near-infrared Fluorescence Cholangiography Assisted Laparoscopic Cholecystectomy Versus Conventional Laparoscopic Cholecystectomy (FALCON): a Multicenter Randomized Controlled Trial. https://clinicaltrials.gov/study/NCT02558556 (accessed (NCT02558556).<br>van den Bos, J.; Schols, R. M.; Boni, L.; Cassinotti, E.; Carus, T.; Luyer, M. D.; Vahrmeijer, A. L.; Mieog, J. S. D.; Warnaar, N.; Berrevoet, F.; et al. Near-infrared fluorescence cholangiography assisted laparoscopic cholecystectomy (FALCON): an international multicentre randomized controlled trial. *Surgical endoscopy* **2023**, *37* (6), 4574-4584. DOI: 10.1007/s00464-023-09935-6.<br>Broderick, R. C.; Lee, A. M.; Cheverie, J. N.; Zhao, B.; Blitzer, R. R.; Patel, R. J.; Soltero, S.; Sandler, B. J.; Jacobsen, G. R.; Doucet, J. J.; Horgan, S. Fluorescent cholangiography significantly improves patient outcomes for laparoscopic cholecystectomy. *Surgical endoscopy* **2021**, *35* (10), 5729-5739. DOI: 10.1007/s00464-020-08045-x<br>Ishizawa, T.; Kaneko, J.; Inoue, Y.; Takemura, N.; Seyama, Y.; Aoki, T.; Beck, Y.; Sugawara, Y.; Hasegawa, K.; Harada, N.; et al. Application of fluorescent cholangiography to single-incision laparoscopic cholecystectomy. *Surgical endoscopy* **2011**, *25* (8), 2631-2636. DOI: 10.1007/s00464-011-1616-2.<br>Ishizawa, T.; Bandai, Y.; Ijichi, M.; Kaneko, J.; Hasegawa, K.; Kokudo, N. Fluorescent cholangiography illuminating the biliary tree during laparoscopic cholecystectomy. *The British journal of surgery* **2010**, *97* (9), 1369-1377. DOI: 10.1002/bjs.7125.<br>Sherwinter, D. A. Identification of anomolous biliary anatomy using near-infrared cholangiography. *Journal of gastrointestinal surgery : official journal of the Society for Surgery of the Alimentary Tract* **2012**, *16* (9), 1814-1815. DOI: 10.1007/s11605-012-1945-z.<br>Dip, F.; LoMenzo, E.; Sarotto, L.; Phillips, E.; Todeschini, H.; Nahmod, M.; Alle, L.; Schneider, S.; Kaja, L.; Boni, L.; et al. Randomized Trial of Near-infrared Incisionless Fluorescent Cholangiography. *Annals of surgery* **2019**, *270* (6), 992-999. DOI: 10.1097/SLA.0000000000003178.
pancreaticoduodenectomy	Hutteman, M.; van der Vorst, J. R.; Mieog, J. S.; Bonsing, B. A.; Hartgrink, H. H.; Kuppen, P. J.; Lowik, C. W.; Frangioni, J. V.; van de Velde, C. J.; Vahrmeijer, A. L. Near-infrared fluorescence imaging in patients undergoing pancreaticoduodenectomy. *European surgical research. Europaische chirurgische Forschung. Recherches chirurgicales europeennes* **2011**, *47* (2), 90-97. DOI: 10.1159/000329411.
acute cholecystitis	Lehrskov, L. L.; Westen, M.; Larsen, S. S.; Jensen, A. B.; Kristensen, B. B.; Bisgaard, T. Fluorescence or X-ray cholangiography in elective laparoscopic cholecystectomy: a randomized clinical trial. *The British journal of surgery* **2020**, *107* (6), 655-661. DOI: 10.1002/bjs.11510.<br>Koong, J. K.; Ng, G. H.; Ramayah, K.; Koh, P. S.; Yoong, B. K. Early identification of the critical view of safety in laparoscopic cholecystectomy using indocyanine green fluorescence cholangiography: A randomised

	controlled study. *Asian journal of surgery* **2021**, *44* (3), 537-543. DOI: 10.1016/j.asjsur.2020.11.002.<br>Di Maggio, F.; Hossain, N.; De Zanna, A.; Husain, D.; Bonomo, L. Near-Infrared Fluorescence Cholangiography can be a Useful Adjunct during Emergency Cholecystectomies. *Surgical innovation* **2022**, *29* (4), 526-531. DOI: 10.1177/1553350620958562.

Table S3. Literature references of ICG for intraoperative tumor "light up"

surgery type	References
lung metastases lesions	Li, H.; Zhou, J.; Chi, C.; Mao, Y.; Yang, F.; Tian, J.; Wang, J. Clinical application of near-infrared thoracoscope with indocyanine green in video-assisted thoracoscopic bullectomy. *Journal of thoracic disease* **2016**, *8* (7), 1841-1845. DOI: 10.21037/jtd.2016.06.02<br>Kasai, Y.; Tarumi, S.; Chang, S. S.; Misaki, N.; Gotoh, M.; Go, T.; Yokomise, H. Clinical trial of new methods for identifying lung intersegmental borders using infrared thoracoscopy with indocyanine green: comparative analysis of 2- and 1-wavelength methods. *European journal of cardio-thoracic surgery : official journal of the European Association for Cardio-thoracic Surgery* **2013**, *44* (6), 1103-1107. DOI: 10.1093/ejcts/ezt168.<br>Pardolesi, A.; Veronesi, G.; Solli, P.; Spaggiari, L. Use of indocyanine green to facilitate intersegmental plane identification during robotic anatomic segmentectomy. *The Journal of thoracic and cardiovascular surgery* **2014**, *148* (2), 737-738. DOI: 10.1016/j.jtcvs.2014.03.001
liver metastases lesions	van der Vorst, J. R.; Schaafsma, B. E.; Hutteman, M.; Verbeek, F. P.; Liefers, G. J.; Hartgrink, H. H.; Smit, V. T.; Lowik, C. W.; van de Velde, C. J.; Frangioni, J. V.; Vahrmeijer, A. L. Near-infrared fluorescence-guided resection of colorectal liver metastases. *Cancer* **2013**, *119* (18), 3411-3418. DOI: 10.1002/cncr.28203<br>Tummers, Q. R.; Verbeek, F. P.; Prevoo, H. A.; Braat, A. E.; Baeten, C. I.; Frangioni, J. V.; van de Velde, C. J.; Vahrmeijer, A. L. First experience on laparoscopic near-infrared fluorescence imaging of hepatic uveal melanoma metastases using indocyanine green. *Surgical innovation* **2015**, *22* (1), 20-25. DOI: 10.1177/1553350614535857<br>Ishizuka, M.; Kubota, K.; Kita, J.; Shimoda, M.; Kato, M.; Sawada, T. Intraoperative observation using a fluorescence imaging instrument during hepatic resection for liver metastasis from colorectal cancer. *Hepato-gastroenterology* **2012**, *59* (113), 90-92. DOI: 10.5754/hge11223<br>Morita, Y.; Sakaguchi, T.; Unno, N.; Shibasaki, Y.; Suzuki, A.; Fukumoto, K.; Inaba, K.; Baba, S.; Takehara, Y.; Suzuki, S.; Konno, H. Detection of hepatocellular carcinomas with near-infrared fluorescence imaging using indocyanine green: its usefulness and limitation. *International journal of clinical oncology* **2013**, *18* (2), 232-241. DOI: 10.1007/s10147-011-0367-3<br>Liu, Y.; Zhao, Y. M.; Akers, W.; Tang, Z. Y.; Fan, J.; Sun, H. C.; Ye, Q. H.; Wang, L.; Achilefu, S. First in-human intraoperative imaging of HCC using the fluorescence goggle system and transarterial delivery of near-infrared

	fluorescent imaging agent: a pilot study. *Translational research : the journal of laboratory and clinical medicine* **2013**, *162* (5), 324-331. DOI: 10.1016/j.trsl.2013.05.002<br>Achterberg, F. B.; Bijlstra, O. D.; Slooter, M. D.; Sibinga Mulder, B. G.; Boonstra, M. C.; Bouwense, S. A.; Bosscha, K.; Coolsen, M. M. E.; Derksen, W. J. M.; Gerhards, M. F.; et al. ICG-Fluorescence Imaging for Margin Assessment During Minimally Invasive Colorectal Liver Metastasis Resection. *JAMA Netw Open* **2024**, *7* (4), e246548. DOI: 10.1001/jamanetworkopen.2024.6548
pancreatic cancers	Hutteman, M.; van der Vorst, J. R.; Mieog, J. S.; Bonsing, B. A.; Hartgrink, H. H.; Kuppen, P. J.; Lowik, C. W.; Frangioni, J. V.; van de Velde, C. J.; Vahrmeijer, A. L. Near-infrared fluorescence imaging in patients undergoing pancreaticoduodenectomy. *European surgical research. Europaische chirurgische Forschung. Recherches chirurgicales europeennes* **2011**, *47* (2), 90-97. DOI: 10.1159/000329411
ovarian cancer	Tummers, Q. R.; Hoogstins, C. E.; Peters, A. A.; de Kroon, C. D.; Trimbos, J. B.; van de Velde, C. J.; Frangioni, J. V.; Vahrmeijer, A. L.; Gaarenstroom, K. N. The Value of Intraoperative Near-Infrared Fluorescence Imaging Based on Enhanced Permeability and Retention of Indocyanine Green: Feasibility and False-Positives in Ovarian Cancer. *PloS one* **2015**, *10* (6), e0129766. DOI: 10.1371/journal.pone.0129766

Table S4. Literature references of ICG for perfusion assessment

surgery type	References
reconstructive surgeries	Roostaeian, J.; Harris, R.; Farkas, J. P.; Barton, F. E.; Kenkel, J. M. Comparison of Limited-Undermining Lipoabdominoplasty and Traditional Abdominoplasty Using Laser Fluorescence Imaging. *Aesthetic surgery journal* **2014**, *34* (5), 741-747. DOI: 10.1177/1090820X14532286<br>Swanson, E. Comparison of Limited and Full Dissection Abdominoplasty Using Laser Fluorescence Imaging to Evaluate Perfusion of the Abdominal Skin. *Plastic and reconstructive surgery* **2015**, *136* (1), 31e-43e. DOI: 10.1097/PRS.0000000000001376<br>Jafari, M. D.; Wexner, S. D.; Martz, J. E.; McLemore, E. C.; Margolin, D. A.; Sherwinter, D. A.; Lee, S. W.; Senagore, A. J.; Phelan, M. J.; Stamos, M. J. Perfusion assessment in laparoscopic left-sided/anterior resection (PILLAR II): a multi-institutional study. *Journal of the American College of Surgeons* **2015**, *220* (1), 82-92 e81. DOI: 10.1016/j.jamcollsurg.2014.09.015
tissue transplantations	Lee, B. T.; Hutteman, M.; Gioux, S.; Stockdale, A.; Lin, S. J.; Ngo, L. H.; Frangioni, J. V. The FLARE intraoperative near-infrared fluorescence imaging system: a first-in-human clinical trial in perforator flap breast reconstruction. *Plastic and reconstructive surgery* **2010**, *126* (5), 1472-1481. DOI: 10.1097/PRS.0b013e3181f059c7

	Hitier, M.; Cracowski, J. L.; Hamou, C.; Righini, C.; Bettega, G. Indocyanine green fluorescence angiography for free flap monitoring: A pilot study. *Journal of cranio-maxillo-facial surgery : official publication of the European Association for Cranio-Maxillo-Facial Surgery* **2016**, *44* (11), 1833-1841. DOI: 10.1016/j.jcms.2016.09.001<br>Jansen, S. M.; de Bruin, D. M.; van Berge Henegouwen, M. I.; Strackee, S. D.; Veelo, D. P.; van Leeuwen, T. G.; Gisbertz, S. S. Can we predict necrosis intra-operatively? Real-time optical quantitative perfusion imaging in surgery: study protocol for a prospective, observational, in vivo pilot study. *Pilot and feasibility studies* **2017**, *3*, 65. DOI: 10.1186/s40814-017-0204-1
colorectal cancer	Cahill, R. A.; O'Shea, D. F.; Khan, M. F.; Khokhar, H. A.; Epperlein, J. P.; Mac Aonghusa, P. G.; Nair, R.; Zhuk, S. M. Artificial intelligence indocyanine green (ICG) perfusion for colorectal cancer intra-operative tissue classification. *The British journal of surgery* **2021**, *108* (1), 5-9. DOI: 10.1093/bjs/znaa004

Table S5. Literature references of clinical trials on 5-ALA

surgery type	References
gliomas	Stummer, W.; Stocker, S.; Wagner, S.; Stepp, H.; Fritsch, C.; Goetz, C.; Goetz, A. E.; Kiefmann, R.; Reulen, H. J. Intraoperative Detection of Malignant Gliomas by 5-Aminolevulinic Acid-induced Porphyrin Fluorescence. *Neurosurgery* **1998**, *42* (3), 518-526<br>Stummer, W.; Pichlmeier, U.; Meinel, T.; Wiestler, O. D.; Zanella, F.; Reulen, H. J. Fluorescence-guided surgery with 5-aminolevulinic acid for resection of malignant glioma: a randomised controlled multicentre phase III trial. *The Lancet. Oncology* **2006**, *7* (5), 392-401. DOI: 10.1016/s1470-2045(06<br>Eicker, S. O.; Floeth, F. W.; Kamp, M.; Steiger, H. J.; Hanggi, D. The impact of fluorescence guidance on spinal intradural tumour surgery. *European spine journal : official publication of the European Spine Society, the European Spinal Deformity Society, and the European Section of the Cervical Spine Research Society* **2013**, *22* (6), 1394-1401. DOI: 10.1007/s00586-013-2657-0<br>Della Puppa, A.; Ciccarino, P.; Lombardi, G.; Rolma, G.; Cecchin, D.; Rossetto, M. 5-Aminolevulinic acid fluorescence in high grade glioma surgery: surgical outcome, intraoperative findings, and fluorescence patterns. *BioMed research international* **2014**, *2014*, 232561. DOI: 10.1155/2014/232561<br>Schupper, A. J.; Baron, R. B.; Cheung, W.; Rodriguez, J.; Kalkanis, S. N.; Chohan, M. O.; Andersen, B. J.; Chamoun, R.; Nahed, B. V.; Zacharia, B. E.; et al. 5-Aminolevulinic acid for enhanced surgical visualization of high-grade gliomas: a prospective, multicenter study. *Journal of neurosurgery* **2022**, *136* (6), 1525-1534. DOI: 10.3171/2021.5.JNS21310<br>Floeth, F. W.; Sabel, M.; Ewelt, C.; Stummer, W.; Felsberg, J.; Reifenberger, G.; Steiger, H. J.; Stoffels, G.; Coenen, H. H.; Langen, K. J. Comparison of

	(18)F-FET PET and 5-ALA fluorescence in cerebral gliomas. *European journal of nuclear medicine and molecular imaging* **2011**, *38* (4), 731-741. DOI: 10.1007/s00259-010-1690-z<br>Valdes, P. A.; Jacobs, V.; Harris, B. T.; Wilson, B. C.; Leblond, F.; Paulsen, K. D.; Roberts, D. W. Quantitative fluorescence using 5-aminolevulinic acid-induced protoporphyrin IX biomarker as a surgical adjunct in low-grade glioma surgery. *Journal of neurosurgery* **2015**, *123* (3), 771-780. DOI: 10.3171/2014.12.JNS14391<br>Hosmann, A.; Millesi, M.; Wadiura, L. I.; Kiesel, B.; Mercea, P. A.; Mischkulnig, M.; Borkovec, M.; Furtner, J.; Roetzer, T.; Wolfsberger, S.; et al. 5-ALA Fluorescence Is a Powerful Prognostic Marker during Surgery of Low-Grade Gliomas (WHO Grade II)-Experience at Two Specialized Centers. *Cancers* **2021**, *13* (11). DOI: 10.3390/cancers13112540<br>Sanai, N.; Snyder, L. A.; Honea, N. J.; Coons, S. W.; Eschbacher, J. M.; Smith, K. A.; Spetzler, R. F. Intraoperative confocal microscopy in the visualization of 5-aminolevulinic acid fluorescence in low-grade gliomas. *Journal of neurosurgery* **2011**, *115* (4), 740-748. DOI: 10.3171/2011.6.JNS11252<br>Lau, D.; Hervey-Jumper, S. L.; Chang, S.; Molinaro, A. M.; McDermott, M. W.; Phillips, J. J.; Berger, M. S. A prospective Phase II clinical trial of 5-aminolevulinic acid to assess the correlation of intraoperative fluorescence intensity and degree of histologic cellularity during resection of high-grade gliomas. *Journal of neurosurgery* **2016**, *124* (5), 1300-1309. DOI: 10.3171/2015.5.JNS1577
glioblastoma	Stummer, W.; Novotny, A.; Stepp, H.; Goetz, C.; Bise, K.; Reulen, H. J. Fluorescence-guided resection of glioblastoma multiforme utilizing 5-ALA-induced porphyrins: a prospective study in 52 consecutive patients. *Journal of neurosurgery* **2000**, *93* (6), 1003-1013<br>Cordova, J. S.; Gurbani, S. S.; Holder, C. A.; Olson, J. J.; Schreibmann, E.; Shi, R.; Guo, Y.; Shu, H. K.; Shim, H.; Hadjipanayis, C. G. Semi-Automated Volumetric and Morphological Assessment of Glioblastoma Resection with Fluorescence-Guided Surgery. *Molecular imaging and biology* **2016**, *18* (3), 454-462. DOI: 10.1007/s11307-015-0900-2
meningioma	Eicker, S. O.; Floeth, F. W.; Kamp, M.; Steiger, H. J.; Hanggi, D. The impact of fluorescence guidance on spinal intradural tumour surgery. *European spine journal : official publication of the European Spine Society, the European Spinal Deformity Society, and the European Section of the Cervical Spine Research Society* **2013**, *22* (6), 1394-1401. DOI: 10.1007/s00586-013-2657-0<br>Cornelius, J. F.; Slotty, P. J.; Kamp, M. A.; Schneiderhan, T. M.; Steiger, H. J.; El-Khatib, M. Impact of 5-aminolevulinic acid fluorescence-guided surgery on the extent of resection of meningiomas--with special regard to high-grade tumors. *Photodiagnosis and photodynamic therapy* **2014**, *11* (4), 481-490. DOI: 10.1016/j.pdpdt.2014.07.008<br>Millesi, M.; Kiesel, B.; Mischkulnig, M.; Martinez-Moreno, M.; Wohrer, A.; Wolfsberger, S.; Knosp, E.; Widhalm, G. Analysis of the surgical benefits of 5-ALA-induced fluorescence in intracranial meningiomas: experience in 204 meningiomas. *Journal of neurosurgery* **2016**, *125* (6), 1408-1419. DOI: 10.3171/2015.12.JNS151513

	Della Puppa, A.; Rustemi, O.; Gioffre, G.; Troncon, I.; Lombardi, G.; Rolma, G.; Sergi, M.; Munari, M.; Cecchin, D.; Gardiman, M. P.; Scienza, R. Predictive value of intraoperative 5-aminolevulinic acid-induced fluorescence for detecting bone invasion in meningioma surgery. *Journal of neurosurgery* **2014**, *120* (4), 840-845. DOI: 10.3171/2013.12.JNS131642
brain metastases	Marhold, F.; Mercea, P. A.; Scheichel, F.; Berghoff, A. S.; Heicappell, P.; Kiesel, B.; Mischkulnig, M.; Borkovec, M.; Wolfsberger, S.; Woehrer, A.; et al. Detailed analysis of 5-aminolevulinic acid induced fluorescence in different brain metastases at two specialized neurosurgical centers: experience in 157 cases. *Journal of neurosurgery* **2019**, 1-12. DOI: 10.3171/2019.6.JNS1997<br>Kamp, M. A.; Grosser, P.; Felsberg, J.; Slotty, P. J.; Steiger, H. J.; Reifenberger, G.; Sabel, M. 5-aminolevulinic acid (5-ALA)-induced fluorescence in intracerebral metastases: a retrospective study. *Acta neurochirurgica* **2012**, *154* (2), 223-228; discussion 228. DOI: 10.1007/s00701-011-1200-5<br>Kamp, M. A.; Munoz-Bendix, C.; Mijderwijk, H. J.; Turowski, B.; Dibue-Adjei, M.; von Sass, C.; Cornelius, J. F.; Steiger, H. J.; Rapp, M.; Sabel, M. Is 5-ALA fluorescence of cerebral metastases a prognostic factor for local recurrence and overall survival? *Journal of neuro-oncology* **2019**, *141* (3), 547-553. DOI: 10.1007/s11060-018-03066-y
breast cancer	Ottolino-Perry, K.; Shahid, A.; DeLuca, S.; Son, V.; Sukhram, M.; Meng, F.; Liu, Z.; Rapic, S.; Anantha, N. T.; Wang, S. C.; et al. Intraoperative fluorescence imaging with aminolevulinic acid detects grossly occult breast cancer: a phase II randomized controlled trial. *Breast Cancer Research* **2021**, *23* (1). DOI: 10.1186/s13058-021-01442-7
bladder cancer	Rolevich, A. I.; Zhegalik, A. G.; Mokhort, A. A.; Minich, A. A.; Vasilevich, V. Y.; Polyakov, S. L.; Krasny, S. A.; Sukonko, O. G. Results of a prospective randomized study assessing the efficacy of fluorescent cystoscopy-assisted transurethral resection and single instillation of doxorubicin in patients with non-muscle-invasive bladder cancer. *World Journal of Urology* **2016**, *35* (5), 745-752. DOI: 10.1007/s00345-016-1927-y

Table S6. Literature references of clinical trials on methylene blue

surgery type	References
parathyroid adenomas	van der Vorst, J. R.; Schaafsma, B. E.; Verbeek, F. P. R.; Swijnenburg, R.-J.; Tummers, Q. R. J. G.; Hutteman, M.; Hamming, J. F.; Kievit, J.; Frangioni, J. V.; van de Velde, C. J. H.; Vahrmeijer, A. L. Intraoperative near-infrared fluorescence imaging of parathyroid adenomas with use of low-dose methylene blue. *Head & Neck* **2014**, *36* (6), 853-858. DOI: https://doi.org/10.1002/hed.23384

	Intraoperative guidance in parathyroid surgery using near-infrared fluorescence imaging and low-dose Methylene Blue. *Surgery* **2015**, *158* (5), 1323-1330. DOI: 10.1016/j.surg.2015.03.027<br>Hillary, S. L.; Guillermet, S.; Brown, N. J.; Balasubramanian, S. P. Use of methylene blue and near-infrared fluorescence in thyroid and parathyroid surgery. *Langenbeck's archives of surgery* **2018**, *403* (1), 111-118. DOI: 10.1007/s00423-017-1641-2<br>Development of a Clinical Protocol to Use Intra-operative Near Infra-red Fluorescent Imaging in Thyroid and Parathyroid Surgery. https://clinicaltrials.gov/study/NCT02089542 (accessed (NCT02089542)
ureteral visualization	Verbeek, F. P.; van der Vorst, J. R.; Schaafsma, B. E.; Swijnenburg, R. J.; Gaarenstroom, K. N.; Elzevier, H. W.; van de Velde, C. J.; Frangioni, J. V.; Vahrmeijer, A. L. Intraoperative near infrared fluorescence guided identification of the ureters using low dose methylene blue: a first in human experience. *The Journal of urology* **2013**, *190* (2), 574-579. DOI: 10.1016/j.juro.2013.02.3187<br>Barnes, T. G.; Hompes, R.; Birks, J.; Mortensen, N. J.; Jones, O.; Lindsey, I.; Guy, R.; George, B.; Cunningham, C.; Yeung, T. M. Methylene blue fluorescence of the ureter during colorectal surgery. *Surgical endoscopy* **2018**, *32* (9), 4036-4043. DOI: 10.1007/s00464-018-6219-8<br>Al-Taher, M.; van den Bos, J.; Schols, R. M.; Bouvy, N. D.; Stassen, L. P. Fluorescence Ureteral Visualization in Human Laparoscopic Colorectal Surgery Using Methylene Blue. *Journal of laparoendoscopic & advanced surgical techniques. Part A* **2016**, *26* (11), 870-875. DOI: 10.1089/lap.2016.0264
neuroendocrine tumor	van der Vorst, J. R.; Vahrmeijer, A. L.; Hutteman, M.; Bosse, T.; Smit, V. T.; van de Velde, C. J.; Frangioni, J. V.; Bonsing, B. A. Near-infrared fluorescence imaging of a solitary fibrous tumor of the pancreas using methylene blue. *World journal of gastrointestinal surgery* **2012**, *4* (7), 180-184. DOI: 10.4240/wjgs.v4.i7.180<br>Tummers, Q. R.; Boonstra, M. C.; Frangioni, J. V.; van de Velde, C. J.; Vahrmeijer, A. L.; Bonsing, B. A. Intraoperative near-infrared fluorescence imaging of a paraganglioma using methylene blue: A case report. *International journal of surgery case reports* **2015**, *6C*, 150-153. DOI: 10.1016/j.ijscr.2014.12.002<br>Galema, H. A.; van Ginhoven, T. M.; Franssen, G. J. H.; Hofland, J.; Bouman, C.; Verhoef, C.; Vahrmeijer, A. L.; Hutteman, M.; Hilling, D. E.; Keereweer, S. Fluorescence-guided surgery using methylene blue to improve identification of metastatic small intestinal neuroendocrine tumours. *The British journal of surgery* **2023**, *110* (5), 541-544. DOI: 10.1093/bjs/znad043
breast cancer	Tummers, Q. R.; Verbeek, F. P.; Schaafsma, B. E.; Boonstra, M. C.; van der Vorst, J. R.; Liefers, G. J.; van de Velde, C. J.; Frangioni, J. V.; Vahrmeijer, A. L. Real-time intraoperative detection of breast cancer using near-infrared fluorescence imaging and Methylene Blue. *European journal of surgical oncology : the journal of the European Society of Surgical Oncology and the British Association of Surgical Oncology* **2014**, *40* (7), 850-858. DOI: 10.1016/j.ejso.2014.02.225

Table S7. Literature references of Clinical trials on IRDye 800CW

No	Conjugated antibody	Target	dose	Date of surgery (after infusion)	NCT No.	References
1	Cetuximab (EGFR targeting)	head & neck cancer (tumor detection & lymph nodes identification)	2.5/25/62.5mg/m^2	3-7 days	NCT01987375	Rosenthal, E. L.; Warram, J. M.; de Boer, E.; Chung, T. K.; Korb, M. L.; Brandwein-Gensler, M.; Strong, T. V.; Schmalbach, C. E.; Morlandt, A. B.; Agarwal, G.; et al. Safety and Tumor Specificity of Cetuximab-IRDye800 for Surgical Navigation in Head and Neck Cancer. *Clinical cancer research : an official journal of the American Association for Cancer Research* **2015**, *21* (16), 3658-3666. DOI: 10.1158/1078-0432.CCR-14-3284<br>Rosenthal, E. L.; Moore, L. S.; Tipirneni, K.; de Boer, E.; Stevens, T. M.; Hartman, Y. E.; Carroll, W. R.; Zinn, K. R.; Warram, J. M. Sensitivity and Specificity of Cetuximab-IRDye800CW to Identify Regional Metastatic Disease in Head and Neck Cancer. *Clinical cancer research : an official journal of the American Association for Cancer Research* **2017**, *23* (16), 4744-4752. DOI: 10.1158/1078-0432.CCR-16-2968<br>Gao, R. W.; Teraphongphom, N.; de Boer, E.; van den Berg, N. S.; Divi, V.; Kaplan, M. J.; Oberhelman, N. J.; Hong, S. S.; Capes, E.; Colevas, A. D.; et al. Safety of panitumumab-IRDye800CW and cetuximab-IRDye800CW for fluorescence-guided surgical navigation in head and neck cancers. *Theranostics* **2018**, *8* (9), 2488-2495. DOI: 10.7150/thno.24487
2		head & neck cancer (tumor detection)	15mg	2 days	NCT03134846	de Wit, J. G.; Vonk, J.; Voskuil, F. J.; de Visscher, S.; Schepman, K. P.; Hooghiemstra, W. T. R.; Linssen, M. D.; Elias, S. G.; Halmos, G. B.; Plaat, B. E. C.; et al. EGFR-targeted fluorescence molecular imaging

						for intraoperative margin assessment in oral cancer patients: a phase II trial. *Nature communications* **2023**, *14* (1), 4952. DOI: 10.1038/s41467-023-40324-8
3		esophageal Cancer (tumor detection)	2.5/25/62.5mg/m^2	2 days	NCT04161560	
4		glioblastoma (tumor detection)	50/100mg	2 days	NCT02855086	Miller, S. E.; Tummers, W. S.; Teraphongphom, N.; van den Berg, N. S.; Hasan, A.; Ertsey, R. D.; Nagpal, S.; Recht, L. D.; Plowey, E. D.; Vogel, H.; et al. First-in-human intraoperative near-infrared fluorescence imaging of glioblastoma using cetuximab-IRDye800. *Journal of neuro-oncology* **2018**, *139* (1), 135-143. DOI: 10.1007/s11060-018-2854-0
5		pancreatic cancer (tumor detection)	50/100mg	2-5 days	NCT02736578	Tummers, W. S.; Miller, S. E.; Teraphongphom, N. T.; Gomez, A.; Steinberg, I.; Huland, D. M.; Hong, S.; Kothapalli, S. R.; Hasan, A.; Ertsey, R.; et al. Intraoperative Pancreatic Cancer Detection using Tumor-Specific Multimodality Molecular Imaging. *Annals of surgical oncology* **2018**, *25* (7), 1880-1888. DOI: 10.1245/s10434-018-6453-2
		rectal Cancer (tumor detection)			NCT04638036	
6	Panitumumab (EGFR targeting)	head & neck cancer (lymph nodes identification)	50mg	1-5 days	NCT02415881	Krishnan, G.; van den Berg, N. S.; Nishio, N.; Juniper, G.; Pei, J.; Zhou, Q.; Lu, G.; Lee, Y. J.; Ramos, K.; Iagaru, A. H.; et al. Metastatic and sentinel lymph node mapping using intravenously delivered Panitumumab-IRDye800CW. *Theranostics* **2021**, *11* (15), 7188-7198. DOI: 10.7150/thno.55389
7					NCT03405142	
8		pancreatic cancer (tumor detection)	25/50/75mg	2-5 days	NCT03384238	Lu, G.; van den Berg, N. S.; Martin, B. A.; Nishio, N.; Hart, Z. P.; van Keulen, S.; Fakurnejad, S.;

						Chirita, S. U.; Raymundo, R. C.; Yi, G.; et al. Tumour-specific fluorescence-guided surgery for pancreatic cancer using panitumumab-IRDye800CW: a phase 1 single-centre, open-label, single-arm, dose-escalation study. *The lancet. Gastroenterology & hepatology* **2020**, *5* (8), 753-764. DOI: 10.1016/S2468-1253(20)30088-1
9		lung cancer (tumor detection)	50mg	1-5 days	NCT03582124	
10		glioma (tumor detection)	50/100mg/kg	1-5 days	NCT03510208	Zhou, Q.; van den Berg, N. S.; Rosenthal, E. L.; Iv, M.; Zhang, M.; Vega Leonel, J. C. M.; Walters, S.; Nishio, N.; Granucci, M.; Raymundo, R.; et al. EGFR-targeted intraoperative fluorescence imaging detects high-grade glioma with panitumumab-IRDye800 in a phase 1 clinical trial. *Theranostics* **2021**, *11* (15), 7130-7143. DOI: 10.7150/thno.60582
11		pediatric neoplasms (brain tumor detection)	0.006/0.25/0.5/1.0 mg/kg	1-5 days	NCT04085887	
12	Bevacizumab (VEGF-A targeting)	breast cancer (tumor detection)	4.5mg	3 days	NCT01508572	Lamberts, L. E.; Koch, M.; de Jong, J. S.; Adams, A. L. L.; Glatz, J.; Kranendonk, M. E. G.; Terwisscha van Scheltinga, A. G. T.; Jansen, L.; de Vries, J.; Lub-de Hooge, M. N.; et al. Tumor-Specific Uptake of Fluorescent Bevacizumab-IRDye800CW Microdosing in Patients with Primary Breast Cancer: A Phase I Feasibility Study. *Clinical cancer research : an official journal of the American Association for Cancer Research* **2017**, *23* (11), 2730-2741. DOI: 10.1158/1078-0432.CCR-16-0437

13		breast cancer (tumor detection)	4.5/10/25/50mg	3 days	NCT02583568	Koller, M.; Qiu, S. Q.; Linssen, M. D.; Jansen, L.; Kelder, W.; de Vries, J.; Kruithof, I.; Zhang, G. J.; Robinson, D. J.; Nagengast, W. B.; et al. Implementation and benchmarking of a novel analytical framework to clinically evaluate tumor-specific fluorescent tracers. *Nature communications* **2018**, *9* (1), 3739. DOI: 10.1038/s41467-018-05727-y
14		Barrett's oesophagus (BE) (early lesion detection)	5 patients: 4.5mg 9 patients: 100μg/ml per cm BE	5 patients: 2 days 9 patients: immediately	NCT02129933	Nagengast, W. B.; Hartmans, E.; Garcia-Allende, P. B.; Peters, F. T. M.; Linssen, M. D.; Koch, M.; Koller, M.; Tjalma, J. J. J.; Karrenbeld, A.; Jorritsma-Smit, A.; et al. Near-infrared fluorescence molecular endoscopy detects dysplastic oesophageal lesions using topical and systemic tracer of vascular endothelial growth factor A. *Gut* **2019**, *68* (1), 7-10. DOI: 10.1136/gutjnl-2017-314953
15		rectal cancer (tumor detection)	4.5mg(first time)+4.5mg(2 days later)	2/3 days after second injection	NCT01972373	Tjalma, J. J. J.; Koller, M.; Linssen, M. D.; Hartmans, E.; de Jongh, S. J.; Jorritsma-Smit, A.; Karrenbeld, A.; de Vries, E. G.; Kleibeuker, J. H.; Pennings, J. P.; et al. Quantitative fluorescence endoscopy: an innovative endoscopy approach to evaluate neoadjuvant treatment response in locally advanced rectal cancer. *Gut* **2020**, *69* (3), 406-410. DOI: 10.1136/gutjnl-2019-319755
16		adenomatous Polyposis (tumor detection)	4.5/10/25mg	3 days	NCT02113202	Hartmans, E.; Tjalma, J. J. J.; Linssen, M. D.; Allende, P. B. G.; Koller, M.; Jorritsma-Smit, A.; Nery, M.; Elias, S. G.; Karrenbeld, A.; de Vries, E. G. E.; et al. Potential Red-Flag Identification of Colorectal Adenomas with Wide-Field Fluorescence Molecular Endoscopy. *Theranostics* **2018**, *8* (6), 1458-1467. DOI: 10.7150/thno.22033

17		pancreatic Cancer (tumor detection)	4.5/10/25mg	3 days	NCT02743975	Mulder, B. G. S.; Koller, M.; Duiker, E. W.; Sarasqueta, A. F.; Burggraaf, J.; Meijer, V. E.; Vahrmeijer, A. L.; Hoogwater, F. J. H.; Bonsing, B. A.; van Dam, G. M.; et al. Intraoperative Molecular Fluorescence Imaging of Pancreatic Cancer by Targeting Vascular Endothelial Growth Factor: A Multicenter Feasibility Dose-Escalation Study. *Journal of nuclear medicine : official publication, Society of Nuclear Medicine* **2023**, *64* (1), 82-89. DOI: 10.2967/jnumed.121.263773
18		pituitary neuroendocrine tumors (tumor detection)	4.5/10/25mg	2-4 days	NCT04212793	Vergeer, R. A.; Postma, M. R.; Schmidt, I.; Korsten-Meijer, A. G.; Feijen, R. A.; Kruijff, S.; Nagengast, W. B.; van Dijk, J. M. C. Detection by fluorescence of pituitary neuroendocrine tumour (PitNET) tissue during endoscopic transsphenoidal surgery using bevacizumab-800CW (DEPARTURE trial): study protocol for a non-randomised, non-blinded, single centre, feasibility and dose-finding trial. **2021**, *11* (10), e049109. DOI: 10.1136/bmjopen-2021-049109
19		cholangiocarcinoma (tumor detection & lymph nodes identification)			NCT03620292	
20		soft-Tissue Sarcomas (tumor detection)	10/25/50mg	3 days	NCT03913806	Steinkamp, P. J.; Pranger, B. K.; Li, M. F.; Linssen, M. D.; Voskuil, F. J.; Been, L. B.; van Leeuwen, B. L.; Suurmeijer, A. J. H.; Nagengast, W. B.; Kruijff, S.; et al. Fluorescence-Guided Visualization of Soft-Tissue Sarcomas by Targeting Vascular Endothelial Growth Factor A: A Phase 1 Single-Center Clinical Trial. *Journal of nuclear medicine :*

						official publication, Society of Nuclear Medicine **2021**, *62* (3), 342-347. DOI: 10.2967/jnumed.120.245696
21		endometriosis (lesions detection)	4.5mg	3 days	NCT02975219	
22		sinonasal inverted papilloma (tumor detection)	10mg	3 days	NCT03925285	Vonk, J.; Voskuil, F. J.; de Wit, J. G.; Heeman, W. T.; Nagengast, W. B.; van Dam, G. M.; Feijen, R. A.; Korsten-Meijer, A.; van der Vegt, B.; Witjes, M. Fluorescence grid analysis for the evaluation of piecemeal surgery in sinonasal inverted papilloma: a proof-of-concept study. *European journal of nuclear medicine and molecular imaging* **2022**, *49* (5), 1640-1649. DOI: 10.1007/s00259-021-05567-x
23	Peptide heterodimer (EGFR and ErbB2 targeting)	Barrett's esophagus(BE) (tumor detection)	topically administered to the distal esophagus using a standard spray catheter		NCT03852576	Chen, J.; Jiang, Y.; Chang, T. S.; Rubenstein, J. H.; Kwon, R. S.; Wamsteker, E. J.; Prabhu, A.; Zhao, L.; Appelman, H. D.; Owens, S. R.; et al. Detection of Barrett's neoplasia with a near-infrared fluorescent heterodimeric peptide. *Endoscopy* **2022**, *54* (12), 1198-1204. DOI: 10.1055/a-1801-2406.<br>(165) A Phase 1 Study to Evaluate the Safety and Feasibility of Intraoperative Detection of Clear Cell Renal Cell Carcinoma Using Indium-111-DOTA-girentuximab-IRDye800CW. https://clinicaltrials.gov/study/NCT02497599 (accessed (NCT02497599)
24	^{111}In-DOTA-Girentuximab (carbonic anhydrase IX targeting)	renal cell carcinoma (tumor detection)	5/10/30/50mg	7 days	NCT02497599	Hekman, M. C.; Rijpkema, M.; Muselaers, C. H.; Oosterwijk, E.; Hulsbergen-Van de Kaa, C. A.; Boerman, O. C.; Oyen, W. J.; Langenhuijsen, J. F.; Mulders, P. F. Tumor-targeted Dual-modality Imaging to Improve Intraoperative Visualization of Clear Cell Renal Cell Carcinoma: A First in Man

						Study. *Theranostics* **2018**, *8* (8), 2161-2170. DOI: 10.7150/thno.23335
25	^{111}In-DOTA-labetuzumab (carcinoembryonic antigen targeting)	colorectal cancer (tumor detection)	2/10/50mg	5/6 days	NCT03699332	de Gooyer, J. M.; Elekonawo, F. M. K.; Bremers, A. J. A.; Boerman, O. C.; Aarntzen, E.; de Reuver, P. R.; Nagtegaal, I. D.; Rijpkema, M.; de Wilt, J. H. W. Multimodal CEA-targeted fluorescence and radioguided cytoreductive surgery for peritoneal metastases of colorectal origin. *Nature communications* **2022**, *13* (1), 2621. DOI: 10.1038/s41467-022-29630-9
26	ABY-029* (EGFR targeting)	soft-tissue sarcoma (tumor detection-ex vivo)	Microdose, 30 nanomoles (237μg)	1-3h	NCT03154411	Samkoe, K. S.; Sardar, H. S.; Gunn, J.; Feldwisch, J.; Linos, K.; Henderson, E.; Pogue, B.; Paulsen, K. Measuring microdose ABY-029 fluorescence signal in a primary human soft-tissue sarcoma resection. *Proceedings of SPIE--the International Society for Optical Engineering* **2019**, *10862*. DOI: 10.1117/12.2510935
27		glioma (tumor detection-ex vivo)	Microdose	1-3h	NCT02901925	
28		head and neck cancer (tumor detection-ex vivo)	30/90/171 nanomoles	1-3h	NCT03282461	Chen, Y.; Streeter, S. S.; Hunt, B.; Sardar, H. S.; Gunn, J. R.; Tafe, L. J.; Paydarfar, J. A.; Pogue, B. W.; Paulsen, K. D.; Samkoe, K. S. Fluorescence molecular optomic signatures improve identification of tumors in head and neck specimens. *Frontiers in medical technology* **2023**, *5*, 1009638. DOI: 10.3389/fmedt.2023.1009638
29	ProstaFluor (Prostate-Specific Membrane	prostate Cancer (tumor detection)			NCT01173146	

	Antigen Targeting)					
30	^{68}Ga-IRDye800CW-BBN (gastrin-releasing peptide receptor (GRPR) targeting)	glioblastoma (tumor detection)	1.0mg	2h	NCT02910804	Li, D.; Zhang, J.; Chi, C.; Xiao, X.; Wang, J.; Lang, L.; Ali, I.; Niu, G.; Zhang, L.; Tian, J.; et al. First-in-human study of PET and optical dual-modality image-guided surgery in glioblastoma using (68)Ga-IRDye800CW-BBN. *Theranostics* **2018**, *8* (9), 2508-2520. DOI: 10.7150/thno.25599
31		low-grade gliomas (tumor detection)	1.0mg	16h	NCT03407781	Chen, L.; Zhang, J.; Chi, C.; Che, W.; Dong, G.; Wang, J.; Du, Y.; Wang, R.; Zhu, Z.; Tian, J.; et al. Lower-grade gliomas surgery guided by GRPR-targeting PET/NIR dual-modality image probe: a prospective and single-arm clinical trial. *Theranostics* **2024**, *14* (2), 819-829. DOI: 10.7150/thno.91554
32	Adalimumab (proinflammatory cytokine tumor necrosis factor α (TNFα) targeting)	Inflammatory bowel disease (IBD) and rheumatoid arthritis (RA) (drug distribution mapping)	4.5/15/25 mg	2 - 4 days	NCT03938701	

Table S8. Literature references of clinical trials on Pafolacianine sodium (OTL38)

surgery type	References
ovarian cancers	Hoogstins, C. E.; Tummers, Q. R.; Gaarenstroom, K. N.; de Kroon, C. D.; Trimbos, J. B.; Bosse, T.; Smit, V. T.; Vuyk, J.; van de Velde, C. J.; Cohen, A. F.; et al. A Novel Tumor-Specific Agent for Intraoperative Near-Infrared Fluorescence Imaging: A Translational Study in Healthy Volunteers and Patients with Ovarian Cancer. *Clinical cancer research : an official journal of the American Association for Cancer Research* **2016**, *22* (12), 2929-2938. DOI: 10.1158/1078-0432.CCR-15-2640<br>Cytalux (pafolacianine) FDA Approval History - Drugs.com. *https://www.drugs.com/history/cytalux.html*<br>A Phase 2, Single Dose, Open-Label Study to Investigate the Safety and Efficacy of OTL38 Injection (OTL38) for Intra-operative Imaging of Folate Receptor-alpha Positive Ovarian Cancer<br>A Phase 3, Randomized, Single Dose, Open-Label Study to Investigate the Safety and Efficacy of OTL38 Injection (OTL38) for Intra-operative Imaging of Folate Receptor Positive Ovarian Cancer. https://clinicaltrials.gov/study/NCT03180307 (accessed (NCT03180307)<br>Randall, L. M.; Wenham, R. M.; Low, P. S.; Dowdy, S. C.; Tanyi, J. L. A phase II, multicenter, open-label trial of OTL38 injection for the intra-operative imaging of folate receptor-alpha positive ovarian cancer. *Gynecologic oncology* **2019**, *155* (1), 63-68. DOI: 10.1016/j.ygyno.2019.07.010<br>Tanyi, J. L.; Randall, L. M. A Phase III Study of Pafolacianine Injection (OTL38) for Intraoperative Imaging of Folate Receptor-Positive Ovarian Cancer (Study 006). **2023**, *41* (2), 276-284. DOI: 10.1200/jco.22.00291
lung cancers	Gangadharan, S.; Sarkaria, I. N.; Rice, D.; Murthy, S.; Braun, J.; Kucharczuk, J.; Predina, J.; Singhal, S. Multiinstitutional Phase 2 Clinical Trial of Intraoperative Molecular Imaging of Lung Cancer. *The Annals of thoracic surgery* **2021**, *112* (4), 1150-1159. DOI: 10.1016/j.athoracsur.2020.09.037<br>A Phase 3, Randomized,Single Dose, Open-Label Study to Investigate the Safety and Efficacy of OTL38 Injection for Intraoperative Imaging of Folate Receptor Positive Lung Nodules. https://clinicaltrials.gov/study/NCT04241315 (accessed (NCT04241315)<br>Sarkaria, I. S.; Martin, L. W.; Rice, D. C.; Blackmon, S. H.; Slade, H. B.; Singhal, S.; Group, E. S. Pafolacianine for intraoperative molecular imaging of cancer in the lung: The ELUCIDATE trial. *The Journal of thoracic and cardiovascular surgery* **2023**, *166* (6), e468-e478. DOI: 10.1016/j.jtcvs.2023.02.025
renal cell carcinoma	Intraoperative Folate Targeted Fluorescence in Renal Cell Carcinoma. https://clinicaltrials.gov/study/NCT02645409 (accessed (NCT02645409)
bladder cancer	Solid Tumor Cancer Surgery With or Without Intraoperative Imaging: A Registry Optional Sub-Study I: ICG Optional Sub-Study II: OTL38. https://clinicaltrials.gov/study/NCT02852252 (accessed (NCT02852252)
pituitary adenoma	A Phase 1, Single Dose, Open-Label Study to Investigate the Safety and Efficacy of OTL38 Injection for Intraoperative Imaging of Folate Receptor-

	alpha Positive Pituitary Adenoma. https://clinicaltrials.gov/study/NCT02629549 (accessed (NCT02629549)
